# Association of antenatal perceived stress and depressive symptoms with birth weight and gestational age: a prospective cohort study within the Action Against Stunting Hub, India

**DOI:** 10.3389/fpubh.2026.1983882

**Published:** 2026-10-27

**Authors:** James Thomas, Sylvia Fernandez Rao, Teena Dasi, Manideep Gunturu, Monica Chilumula, Little Flower Augustine, Hemant Mahajan, Hilary Davies-Kershaw, Claire Heffernan, Ajantha Rudhra, Santosh Kumar Banjara, Sairam Challa, Bharati Kulkarni, Raghu Pullakhandam, Ravindranadh Palika

**Affiliations:** 1 ICMR-National Institute of Nutrition, Indian Council of Medical Research, Hyderabad, India; 2 Department of Population Health, London School of Hygiene and Tropical Medicine, London, United Kingdom

**Keywords:** antenatal depressive symptoms, antenatal perceived stress, birth weight, gestational age, low birth weight, maternal mental health, pregnancy outcomes, preterm birth

## Abstract

**Background:**

Evidence on the association of pregnancy specific perceived stress and depressive symptoms with adverse birth outcomes, including preterm birth (PTB) and low birth weight (LBW), remains limited and inconsistent in India.

**Objective:**

This study aimed to estimate the prevalence of elevated perceived stress and elevated depressive symptoms during the third trimester of pregnancy and examine their associations with birth weight, gestational age, low birth weight (LBW), and preterm birth (PTB) in an Indian cohort.

**Methods:**

A prospective cohort study was conducted within the Action Against Stunting Hub mother child cohort in Hyderabad, India between June 2021 and December 2022. Of the 717 pregnant women enrolled in their third trimester, 616 with available psychosocial data were included in the analysis. Perceived stress, depressive symptom scores and social support were assessed using Perceived Stress Scale 10(PSS-10), Edinburgh Postnatal Depression Scale (EPDS) and Multidimensional Scale for Perceived Social Support (MSPSS) respectively. Gestational age was determined using a hierarchical approach and birth weight was measured within 12 h of delivery. Associations between maternal perceived stress and depressive symptoms scores with birth outcomes were examined using descriptive statistics and multivariable linear and logistic regression analyses (*p* < 0.05).

**Results:**

The prevalence of elevated perceived stress and elevated depressive symptoms among pregnant women during the third trimester was 17.4 and 21.4%, respectively. After adjusting for covariates, each one-point increase in maternal perceived stress score (β = −5.84 g (95%CI −11.49, −0.19, *p* = 0.04)) and depressive symptom scores (β = −7.45g (95%CI −13.72, −1.17, *p* = 0.02)) was significantly associated with lower birth weight, but not with gestational age. However, no association between maternal perceived stress and depressive symptom scores was observed with either LBW and PTB using multivariable logistic regression.

**Conclusion:**

Third trimester maternal perceived stress and depressive symptom scores were associated with modest reductions in birth weight but not with gestational age, LBW, or PTB. Although the observed effect on birth weight was small, these findings highlight the importance of recognizing and addressing maternal psychological well being during antenatal care.

## Introduction

Birth weight and gestational age at delivery are critical indicators of neonatal survival, neurodevelopment, and long term health trajectories. Low birth weight (LBW, < 2,500 g) and (PTB, < 37 completed weeks) are distinct but frequently co-occurring conditions, each arising from premature delivery, impaired fetal growth, or both. Globally, LBW affects approximately 14.6% of live births and PTB 9.9%, with South Asia contributing a substantial share of this burden (1, 2). India exceeds these averages, with LBW prevalence estimated at 17.29% and PTB at 13% (1, 2), contributing disproportionately to neonatal and infant mortality (3) and leading to elevated risk of neurodevelopmental impairment and cardiometabolic disease in later life (4, 5).

Maternal undernutrition, teenage pregnancies, anemia, micronutrient deficiencies particularly iron, folate, and zinc and adverse obstetric history are well established contributors to fetal growth restriction and preterm delivery. Quality of antenatal care also matters. Early registration, adequate visit frequency, appropriate supplementation, and accurate gestational age assessment have each been associated with better birth outcomes (6). However, these factors do not fully explain the variation observed across and within populations, suggesting that other influences on maternal health need to be considered.

Maternal psychological distress during pregnancy has received increasing attention in this context. During the antenatal period, women experience significant hormonal, physiological, and biochemical changes, which may be accompanied by inadequate emotional and social support from family and immediate social environment (7, 8). These multifaceted changes predispose pregnant women to psychological distress. Among the most commonly reported mental health concerns during pregnancy are perceived stress and depressive symptoms (9, 10). A recent systematic review reported that, the pooled prevalence of depressive symptoms and perceived stress symptoms during antenatal period was 27 and 26%, respectively (11). However, the burden of psychological depressive symptoms may vary across populations and trimesters due to differences in cultural and social support practices particularly in low and middle income countries (12).

It has been hypothesized that maternal perceived stress and depressive symptoms may affect fetal growth and development through activation of the hypothalamic pituitary adrenal (HPA) axis and through behavioral mechanisms (13, 14). Several studies have reported that perceived stress and depressive symptoms during pregnancy are associated with adverse birth outcomes including LBW and PTB (12, 15–17). A meta-analysis of 23 studies reported increased risks of PTB (RR 1.35), LBW (RR 1.86), and intrauterine growth restriction (RR 4.39) among women experiencing psychological distress (15). In contrast, studies conducted among women with full term, medically low risk pregnancies have reported an association between antenatal depressive symptoms and higher birth weight in term infants (18). However, few studies have reported no significant association between maternal perceived stress or depressive symptoms and adverse birth outcomes (19–22). Further, a previous study reported that the risk of PTB associated with antenatal depressive symptoms varied across racial/ethnic groups (23). These inconsistent findings may reflect differences in population characteristics, socioeconomic conditions, timing of assessment, and confounding factors. Despite the high burden of LBW and PTB in India, evidence on the effect of antenatal perceived stress and depressive symptoms on birth outcomes remains limited and inconsistent.

Therefore, this study aimed to assess maternal psychological perceived stress and depressive symptom scores during the third trimester of pregnancy and examine their associations with the risk of LBW and PTB in an Indian cohort.

## Methods

### Maternal psychological and social support assessments

This study was nested within the prospective mother child dyad cohort established as a part of Action Against Stunting Hub project in Hyderabad, Telangana, India. Pregnant women residing in the urban communities of Addagutta, Seethaphalmandi, and Warasiguda were recruited between June 2021 and December 2022. A total of 717 women in their third trimester of pregnancy, were enrolled following written informed consent. Apparently healthy pregnant women with a singleton live fetus in the third trimester, residing within a 20 km radius of ICMR-NIN, Hyderabad, and willing to be available for follow up over a period of 2 years were included in the study. Pregnant women with bad obstetric history and gestational diabetes in previous pregnancy were excluded.

Participants and their infants were prospectively followed until 24 months after delivery. The study design and protocols have been described previously (24). During the third trimester, trained field investigators collected electronic data on sociodemographic characteristics, anthropometry, and reproductive health using a structured questionnaire administered face to face in the participant's preferred local language, following standardized procedures to minimize information bias. (Figure 1). All deliveries occurred in institutional settings and birth weights were obtained from official birth reports. The mothers and infants were visited by the field study teams after delivery to collect the additional details about the birth. The study protocol was approved by the Institutional Ethics Committee of the National Institute of Nutrition (ICMR)-Ministry of Health and Family Welfare, Government of India (CR/04/I/2021) and the London School of Hygiene and Tropical Medicine (17915/RR/17513). All study procedures were conducted in accordance with the principles of the Declaration of Helsinki, and confidentiality of participant information was maintained throughout the study.

**Figure 1:**
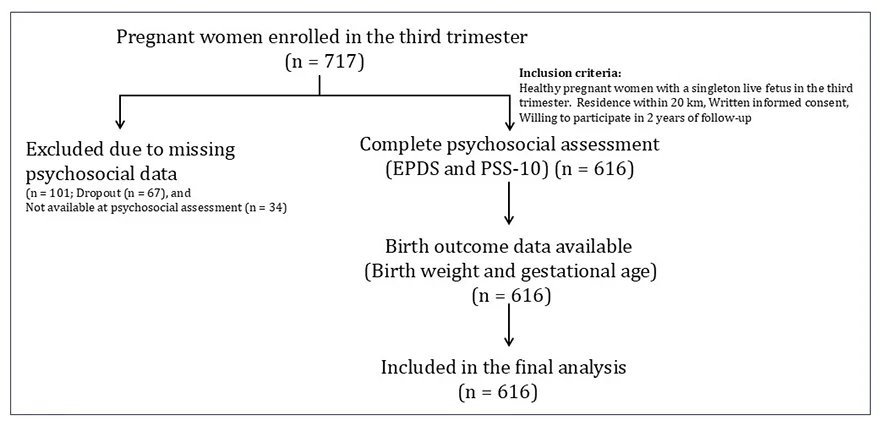
Flow diagram of participant recruitment and inclusion in the final analysis.

### Sample size and statistical power

This analysis was nested within the prospective mother–child cohort of the Action Against Stunting Hub project, and no separate a priori sample-size calculation was undertaken specifically for the present analysis. The analytical sample therefore comprised all participants with available antenatal psychological assessment and birth-outcome data (*n* = 562). As an exploratory assessment, post-hoc statistical power based on the observed adjusted effect estimates was approximately 49 and 60% for the associations of maternal stress and depressive symptom scores, respectively, with birth weight. Given the limitations of post-hoc power calculations, statistical precision was primarily evaluated using effect estimates and 95% confidence intervals.

### Assessment of psychosocial factors (exposures)

Maternal psychosocial wellbeing during pregnancy was assessed using validated instruments measuring depressive symptoms, perceived stress, and social support (24). All questionnaires were translated into local languages (Telugu and Hindi) using standardized forward backward translation procedures and pilot tested before implementation (25). Field investigators underwent rigorous training in questionnaire administration, scoring procedures, and participant privacy. Interviews were conducted in private settings either at participants homes or field offices.

### Maternal depressive symptoms

Depressive symptoms during pregnancy were screened using the Edinburgh Postnatal Depression Scale (EPDS) (26), a validated 10-item screening instrument. Individual items were scored from 0 to 3, yielding a total depressive symptom score ranging from 0 to 30, with higher scores indicating greater symptom severity. Participants with EPDS scores ≥10 were classified as having elevated depressive symptoms (binary variable 0 = no, 1 = yes). The seven negatively worded items (Items 3 and 5–10) were reverse scored before calculating the total EPDS score. No clinical diagnosis of depression was made. In the present study, the internal consistency was 0.83 (Cronbach's α) (27).

### Maternal perceived stress

Perceived stress was assessed using the 10-item Perceived Stress Scale-10 (PSS-10), which measures the degree to which situations in an individual's life are perceived as stressful during the preceding month. Responses were scored on a five point Likert scale ranging from 0 (“never”) to 4 (“very often”), producing a total perceived stress score ranging from 0 to 40. Four positively worded items were reverse coded before score calculation. Participants with perceived stress scores ≥14 were classified as having elevated perceived stress (categorical variable, 0: < 14, 1: ≥14). Internal consistency of the scale was acceptable (Cronbach's α = 0.74) (28).

### Maternal social support

Perceived social support was assessed using the Multidimenstional Scale for Perceived Social Support (MSPSS) (29). The instrument consists of 12 items measuring support received from family, friends, and significant others. Responses were scored on a seven point Likert scale, with higher scores indicating greater perceived social support. Mean scores below 3 were considered indicative of low social support (binary 0 ≥ 3, 1 = less than 3). In the present study, the internal consistency was 0.84 (Cronbach's α).

### Gestational age and birth information

Gestational age at delivery was determined using a hierarchical approach to ensure maximum accuracy. First trimester ultrasonography estimates (*n* = 479) were given highest priority. In the absence of first trimester scans, second trimester ultrasonography estimates (*n* = 81) were used. For participants without ultrasound data, gestational age was calculated from the reported first day of the last menstrual period (*n* = 56). Birth weight was measured within 12 h of delivery by trained hospital staff using calibrated digital infant weighing scales (30). LBW was defined as birth weight < 2,500 g (binary 0: ≥2,500 g, 1: < 2,500 g). PTB was defined as delivery before 37 completed weeks of gestation (binary 0: ≥37 weeks, 1: < 37 weeks).

### Statistical analysis

Statistical analyses were performed using Python (version 3.12.10). Data management and descriptive analyses were conducted using the pandas and NumPy libraries. Continuous variables were expressed as mean and standard deviation (SD), while categorical variables were expressed as frequencies. Continuous variables were categorized and categorical variables were summarized using frequencies and proportions. Normally distributed variables were analyzed using parametric tests, whereas non normally distributed variables were analyzed using non parametric tests. Statistical tests were performed using the SciPy library, including the independent-samples *t*-test, Mann–Whitney *U*-test, chi-square test, Fisher's exact test, and Spearman's rank correlation. The associations between birth weight, maternal perceived stress, depressive symptom scores, social support score and gestational age were assessed using Spearman's rank correlation. Univariable linear regression analyses were performed to assess the association between each independent variable and the continuous outcomes of birth weight or gestational age. Subsequently, separate multivariable linear regression models were constructed to assess the adjusted effects of maternal perceived stress and depressive symptoms on birth weight and gestational age after controlling for potential confounders. Logistic regression models were constructed to assess the adjusted effects of maternal perceived stress and depressive symptoms on LBW and PTB after controlling for potential confounders. Adjusted odds ratios (AORs) and 95% confidence intervals (CIs) were reported (31). Model assumptions, including multicollinearity and goodness of fit, were assessed prior to interpretation. Statistical significance was defined as *p*-value < 0.05.

## Results

Maternal and socio demographic characteristics are presented in Table 1. Of the 616 pregnant women included in the analysis, the majority were aged 18–29 years (79.6%), had secondary or higher education (84.8%), and were homemakers (89.8%). Nearly half completed more than five antenatal visits and primigravida women accounted for 39.5% of participants. Maternal age, education, occupation, gravidity, parity, history of miscarriage or stillbirth, dietary pattern, substance use, and sanitation facility were not significantly associated with either LBW or PTB (*p* > 0.05). LBW, but not PTB was significantly higher among infants whose mothers had 6–10 antenatal visits and among female infants. Both LBW (*p* = 0.013) and PTB (*p* = 0.0035) were significantly more common in deliveries attended by nurses/midwives. PTB was also significantly higher in cesarean section deliveries (*p* = 0.0002).

**Table 1:** Characteristics of mother and neonates.

Maternal characteristics	Total *n* (%)	Low birth weight (LBW) *n* (%)	Normal birth weight (NBW) *n* (%)	@*p*-value	Preterm birth (PTB) *n* (%)	Term birth *n* (%)	#*p*-value
**Age (*****n*** **=** **616)**^**$**^		109 (17.69)	507 (82.31)	100 (16.23)	516 (83.76)
< 18	5 (0.8)	1 (0.9)	4 (0.8)	0.2038	2 (2.0)	3 (0.6)	0.1945
18–23	189 (30.7)	31 (28.5)	158 (31.2)		33 (33.0)	156 (30.2)	
24–29	301 (48.9)	48 (44.0)	253 (49.9)		42 (42.0)	259 (50.2)	
>29	121 (19.6)	29 (26.6)	92 (18.1)		23 (23.0)	98 (19.0)	
Education (*n* = 614)							
No education	38 (6.2)	7 (6.5)	31 (6.1)	0.5787	8 (8.0)	30 (5.8)	0.8096
Primary	55 (9.0)	13 (12.0)	42 (8.3)		9 (9.0)	46 (9.0)	
Secondary	346 (56.3)	61 (56.5)	285 (56.3)		53 (53.0)	293 (57.0)	
Higher	175 (28.5)	27 (25.0)	148 (29.3)		30 (30.0)	145 (28.2)	
Occupation (*n* = 616)							
Not working/home maker	553 (89.8)	100 (91.7)	453 (89.3)	0.5659	94 (94.0)	459 (89.0)	0.1789
Working	63 (10.2)	9 (8.3)	54 (10.7)		6 (6.0)	57 (11.0)	
Previous miscarriage (*n* = 616)							
No	484 (78.6)	84 (77.1)	400 (78.9)	0.7687	74 (74.0)	410 (79.5)	0.2783
Yes	132 (21.4)	25 (22.9)	107 (21.1)		26 (26.0)	106 (20.5)	
Previous stillbirth (*n* = 616)^$^							
No	597 (96.9)	104 (95.4)	493 (97.2)	0.3553	99 (99.0)	498 (96.5)	0.3384
Yes	19 (3.1)	5 (4.6)	14 (2.8)		1 (1.0)	18 (3.5)	
Antenatal visits (*n* = 604)							
≤ 5	276 (45.7)	40 (37.4)	236 (47.5)	0.0493	47 (47.5)	229 (45.4)	0.888
6–10	286 (47.3)	62 (57.9)	224 (45.1)		46 (46.5)	240 (47.5)	
≥11	42 (7.0)	5 (4.7)	37 (7.4)		6 (6.0)	36 (7.1)	
Gravidity (*n* = 616)^$^							
1	243 (39.5)	40 (36.7)	203 (40.0)	0.7497	34 (34.0)	209 (40.5)	0.3945
2–4	358 (58.1)	66 (60.5)	292 (57.6)		63 (63.0)	295 (57.2)	
≥5	15 (2.4)	3 (2.8)	12 (2.4)		3 (3.0)	12 (2.3)	
Parity (*n* = 616)^$^							
Nulliparous	283 (45.9)	49 (44.9)	234 (46.1)	0.9053	40 (40.0)	243 (47.1)	0.2825
1–2	313 (50.8)	56 (51.4)	257 (50.7)		58 (58.0)	255 (49.4)	
3 or more	20 (3.3)	4 (3.7)	16 (3.2)		2 (2.0)	18 (3.5)	
Birth attendant (*n* = 578)^$^							
Doctor	476 (82.4)	76 (73.8)	400 (84.2)	0.013	85 (92.4)	391 (80.5)	0.0035
Nurse/midwife	99 (17.1)	25 (24.3)	74 (15.6)		6 (6.5)	93 (19.1)	
Others	3 (0.5)	2 (1.9)	1 (0.2)		1 (1.1)	2 (0.4)	
Mode of delivery (*n* = 578)							
C-section	276 (47.8)	40 (38.8)	236 (49.7)	0.0588	61 (66.3)	215 (44.2)	0.0002
Vaginal	302 (52.2)	63 (61.2)	239 (50.3)		31 (33.7)	271 (55.8)	
Dietary pattern (*n* = 614)^$^							
Non-vegetarian	598 (97.4)	106 (98.2)	492 (97.2)	1	98 (100.0)	500 (96.9)	0.3415
Vegetarian	9 (1.5)	1 (0.9)	8 (1.6)		0 (0.0)	9 (1.7)	
Others	7 (1.1)	1 (0.9)	6 (1.2)		0 (0.0)	7 (1.4)	
Tobacco use during pregnancy (*n* = 616)^$^							
No	613 (99.5)	108 (99.1)	505 (99.6)	0.443	98 (98.0)	515 (99.8)	0.0701
Yes	3 (0.5)	1 (0.9)	2 (0.4)		2 (2.0)	1 (0.2)	
Alcohol use during pregnancy (*n* = 616)^$^							
Yes	123 (20.0)	21 (19.3)	102 (20.1)	0.9138	25 (25.0)	98 (19.0)	0.3077
No	492 (79.9)	88 (80.7)	404 (79.7)		75 (75.0)	417 (80.8)	
Prefer not to answer	1 (0.1)	0 (0.0)	1 (0.2)		0 (0.0)	1 (0.2)	
Sanitation facility (*n* = 616)^$^							
Flush elsewhere	8 (1.3)	1 (0.9)	7 (1.4)	0.3797	2 (2.0)	6 (1.2)	0.533
Flush sewer/septic	82 (13.3)	16 (14.7)	66 (13.0)		10 (10.0)	72 (13.9)	
Open land/water	1 (0.2)	0 (0.0)	1 (0.2)		0 (0.0)	1 (0.2)	
Pit latrine	495 (80.3)	83 (76.1)	412 (81.3)		85 (85.0)	410 (79.5)	
Shared	30 (4.9)	9 (8.3)	21 (4.1)		3 (3.0)	27 (5.2)	
Infant gender (*n* = 616)							
Female	308 (50.0)	42 (38.5)	266 (52.5)	0.0113	50 (50.0)	258 (50.0)	1
Male	308 (50.0)	67 (61.5)	241 (47.5)		50 (50.0)	258 (50.0)	

LBW, low birth weight (< 2.5 kg) and NBW, normal birth weight (≥2.5 kg). Gestational age was categorized as preterm (< 37 weeks) and term (≥37 weeks). Vertical percentages were calculated within outcome categories (LBW/NBW and preterm/term). Categorical variables were compared using the chi-square test and $ fisher exact test; ^@^indicates *p*-values for comparison between low birth weight and normal birth weight; ^#^indicates *p*-values for comparison between preterm and term births. A *p*-value < 0.05 was considered statistically significant.

The prevalence of elevated perceived stress and depressive symptoms among the study participants was 17.4 and 21.4% respectively (Table 2). Nearly 98% women reported receiving social support from their family and friends during pregnancy. Mean birth weight and gestational age did not differ significantly between pregnant women with and without elevated perceived stress (2.73 vs. 2.77 kgs; *p* = 0.37 and 38.05 vs. 38.28 weeks; *p* = 0.35), pregnant women with and without elevated depressive symptoms(2.73 vs. 2.77 kgs; *p* = 0.25 and 38.25 vs. 38.23 weeks; *p* = 0.93) or between women who received social support and those who did not(2.76 vs. 2.67 kgs; *p* = 0.57 and 38.21 vs. 38.21 weeks; *p* = 0.99). In contrast, preterm infants had significantly lower birth weight and gestational age compared to term infants (2.50 vs. 2.81 kg and 35.53 vs. 38.76 weeks; *p* < 0.001). Birth weight and gestational age were negatively correlated with perceived stress scores (Figure 2A) and positively correlated with social support (Figure 2C). In contrast, depressive symptom scores were positively correlated with gestational age and negatively correlated with birth weight (Figure 2B). However, none of these correlations were statistically significant. Gestational age was significantly positively correlated with birth weight. (*r* = 0.279, *p* < 0.001) (Figure 2D).

**Table 2:** Comparison of mean birth weight and mean gestational age according to maternal psychosocial factors during pregnancy.

Condition	*n* (%)	Birth weight (kg) (mean ±SD)	*p*-value (BWt)	Gestational age (weeks) (mean ±SD)	*p*-value (GA)
Screened positive for perceived Stress (≥14)	107 (17.4)	2.73 ± 0.44	0.3703	38.05 ± 2.48	0.3545
Without perceived stress (< 14)	509 (82.6)	2.77 ± 0.43		38.28 ± 1.52	
Screened positive for Depressive symptoms (≥10)	132 (21.4)	2.73 ± 0.42	0.2555	38.25 ± 1.83	0.9343
Without depressive symptoms (< 10)	484 (78.6)	2.77 ± 0.43		38.23 ± 1.70	
Low social support (< 3)	12 (2.1)	2.67 ± 0.51	0.5681	38.21 ± 1.98	0.9988
Moderate/high social support (≥3)	563 (97.9)	2.76 ± 0.42		38.21 ± 1.71	
Preterm birth (< 37 weeks)	100 (16.2)	2.50 ± 0.46	< 0.001	35.53 ± 1.85	< 0.001
Term birth (≥37 weeks)	516 (83.8)	2.81 ± 0.41		38.76 ± 1.09	

Mean birth weight and gestational age were compared between groups using independent samples t-test. Values are presented as mean ± SD with corresponding *p*-values. A *p*-value < 0.05 was considered statistically significant.

**Figure 2:**
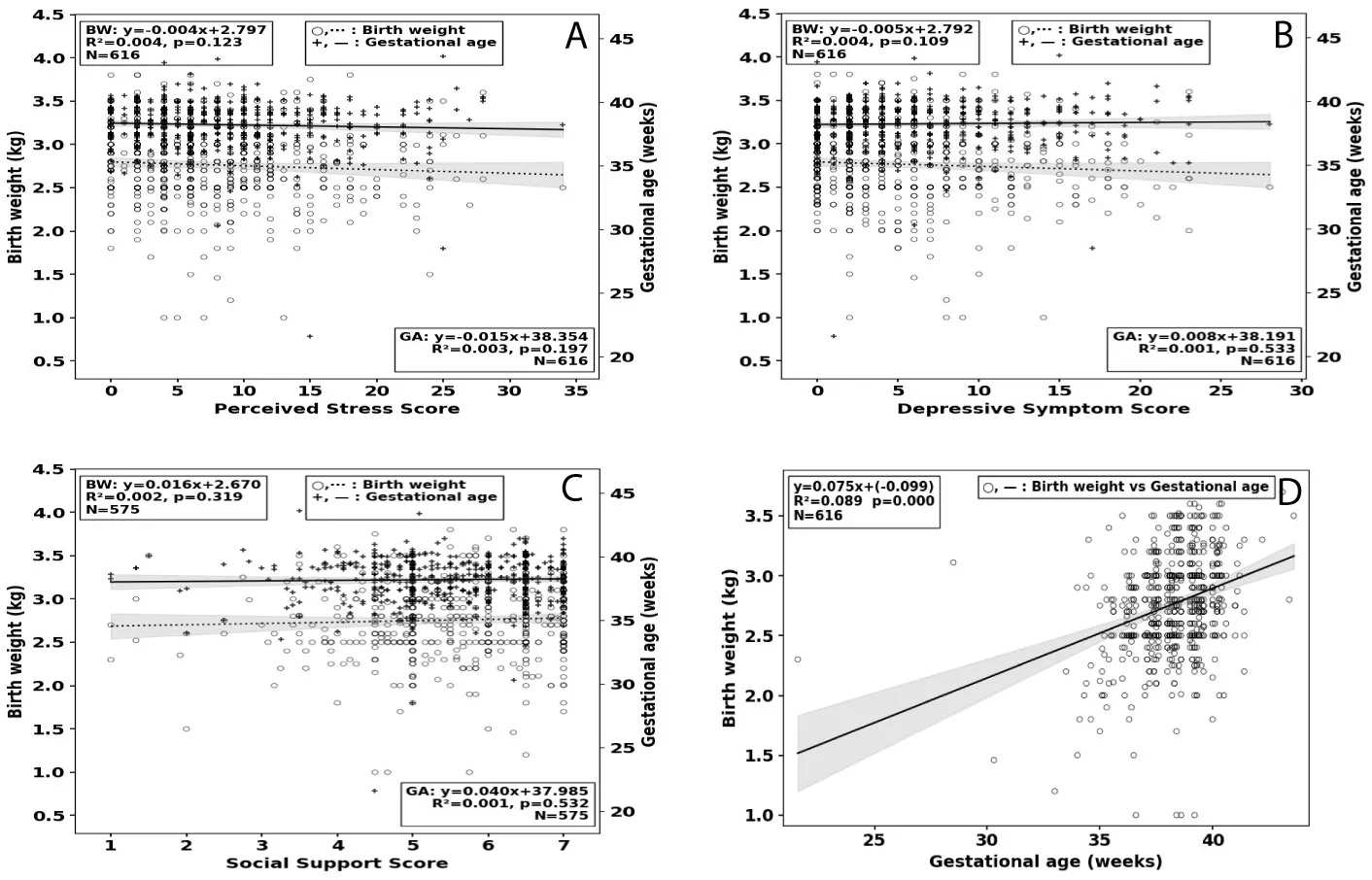
Correlation of maternal psychosocial scores with birth weight and gestational age: Dual axis scatter plots showing the relationship between antenatal perceived stress score **(A)**, depressive symptom score **(B)** and social support score **(C)** with birth weight and gestational age among pregnant women. Panel **(D)** shows the association between gestational age and birth weight.

In univariable analysis (Table 3), each independent variable was examined separately as a predictor of birth weight and gestational age. Gestational age (β = 74.83) and male gender (β = 137.63), were significantly associated with higher birth weight. In contrast, birth weight (β = 0.0012) and working women (β = 0.56) were significantly positively associated with higher gestational age. However, prenatal perceived stress score, depressive symptom score or social support score were not significantly associated with birth weight or gestational age.

**Table 3:** Univariable regression analysis.

Predictor	Variable	Dependent variable : birth weight (g)		Dependent variable : gestational age (weeks)	
		Univariable		Univariable	
		β (95% CI)	*p*-value	β (95% CI)	*p*-value
Perceived stress score	Perceived stress score	−4.37 (−9.93, 1.19)	0.123	−0.01 (-0.04, 0.01)	0.197
Depressive symptom score	Depressive symptoms score	−5.24 (−11.65, 1.17)	0.109	0.01 (-0.02, 0.03)	0.533
Social support	Social support	15.86 (−15.34, 47.05)	0.319	0.04 (-0.09, 0.17)	0.532
Gestational age	Gestational age	74.83 (55.87, 93.8)	< 0.001	–
Birth weight	Birth weight	–	–	0.0012 (0.0, 0.0015)	< 0.001
Age	18–23 years (ref)	–	–	0 (ref)	–
	24–29 years	39.89 (−38.2, 117.99)	0.316	0.08 (−0.24, 0.39)	0.63
	>29 years	−27.59 (−125.85, 70.67)	0.582	−0.13 (−0.53, 0.26)	0.505
Gravidity	1 (ref)	–	–	0 (ref)	–
	2–4	15.2 (−55.36, 85.76)	0.672	−0.23 (−0.51, 0.05)	0.113
	≥5	136.5 (−89.34, 362.35)	0.236	0.11 (−0.79, 1.01)	0.804
Parity	Nulliparous (ref)	–	–	0 (ref)	–
	1–2	16.33 (−53.35, 86.0)	0.646	−0.23 (−0.51, 0.05)	0.101
	3 or more	72.84 (−123.7, 269.38)	0.467	0.51 (−0.27, 1.29)	0.199
Antenatal visits	< =5 (ref)	0 (ref)	–
	6–10	−31.2 (−103.05, 40.66)	0.394	−0.01 (−0.29, 0.28)	0.954
	≥11	77.61 (−63.43, 218.66)	0.28	0.18 (−0.39, 0.74)	0.54
Gender	Female (ref)	0 (ref)	–
	Male	137.63 (70.07, 205.19)	< 0.001	−0.07 (−0.34, 0.2)	0.604
Education	Higher (ref)	0 (ref)	–
	No education	0.57 (−149.33, 150.46)	0.994	−0.37 (−0.98, 0.23)	0.23
	Primary	−120.39 (−249.87, 9.08)	0.068	0.1 (−0.43, 0.62)	0.715
	Secondary	0.41 (−77.28, 78.1)	0.992	0.22 (−0.09, 0.53)	0.169
Occupation	Not working/home maker (ref)	0 (ref)	–
	Working	50.28 (−62.57, 163.13)	0.382	0.56 (0.11, 1.0)	0.015
Previous stillbirth	No (ref)	0 (Ref)	–
	Yes	−61.59 (−259.43, 136.25)	0.541	0.14 (−0.65, 0.93)	0.726
Previous miscarriage	No (ref)	0 (ref)	–
	Yes	50.75 (−32.54, 134.04)	0.232	−0.01 (−0.35, 0.32)	0.937

Univariable regression analysis examining the associations of psychosocial factors and maternal characteristics with birth weight and gestational age among pregnant women. Regression coefficients (β), 95% confidence intervals (CI), and *p*-values are presented.

Separate multivariable linear regression models were constructed to assess the associations of maternal perceived stress and depressive symptom scores with birth weight and gestational age. After adjustment for covariates each one-point increase in maternal perceived stress score (model-1) and depressive symptom scores (model-2) was significantly associated with LBW (perceived stress: β = −5.84g, (95% CI: −11.49, −0.19); *p* = 0.04; depressive symptoms: β = −7.45g, (95% CI: −13.72, −1.17); *p* = 0.02) (Table 4). In contrast, neither maternal perceived stress (model-3) nor depressive symptom score (model-4) was significantly associated with gestational age. However, in logistic regression analysis, neither perceived stress nor depressive symptom score were significantly associated with LBW (perceived stress AOR: 1.03; 95% CI: 0.99, 1.07; depressive symptoms AOR: 1.03; 95% CI: 0.98, 1.07) or PTB (perceived stress AOR: 1.02; 95% CI: 0.98, 1.06; depressive symptoms AOR: 1.01; 95% CI: 0.96, 1.05). Overall, third trimester maternal perceived stress and depressive symptom scores were significantly associated with reduced birth weight in continuous analyses; however, no significant associations were observed for categorical outcomes of LBW or PTB.

**Table 4:** Multivariable regression analysis.

	Birth weight (g)		Gestational age (weeks)	
Multivariable linear regression (*N* = 562)	Model 1 perceived stress score β (95% CI) (*R*^2^ = 0.146)	Model 2 depressive symptoms score β (95% CI) (*R*^2^ = 0.148)	Model 3 perceived stress score β (95% CI) (*R*^2^ = 0.038)	Model 4 depressive symptoms β (95% CI) (*R*^2^ = 0.034)
	−5.84^*^ (−11.49, −0.19)	−7.45^*^ (−13.72, −1.17)	−0.02 (−0.04, 0.00)	0.01 (−0.02, 0.04)
**For LBW**		**For PTB**	
Logistic regression (*N* = 562)	Perceived stress score AOR(95% CI) (*R*^2^ = 0.104)	Depressive symptoms score AOR(95% CI) (*R*^2^ = 0.102)	Perceived stress score AOR(95% CI) (*R*^2^ = 0.026)	Depressive symptom score AOR(95% CI) (*R*^2^ = 0.025)
	1.03 (0.99, 1.07)	1.03 (0.98, 1.07)	1.02 (0.98, 1.06)	1.01 (0.96, 1.05)

Multivariable linear and logistic regression analyses examining the associations of maternal antenatal perceived stress and depressive symptoms with birth weight, LBW, gestational age, and PTB among pregnant women (analytical sample, *n* = 562). For both linear and logistic regression analyses, perceived stress score and depressive symptom score were used as continuous exposure variables. Birth weight and gestational age were continuous outcomes in the linear regression models, whereas LBW/normal birth weight and PTB/term birth were binary outcomes in the logistic regression models. Model 1 assessed the association of perceived stress score with birth weight and LBW; Model 2 assessed the association of depressive symptom score with birth weight and LBW; Model 3 assessed the association of perceived stress score with gestational age and PTB; and Model 4 assessed the association of depressive symptom score with gestational age and PTB. All models were adjusted for maternal age, number of antenatal visits, maternal education, infant sex, maternal occupation, parity, previous miscarriage, previous stillbirth, and perceived social support. Models 1 and 2 were additionally adjusted for gestational age at delivery, whereas model 3 and 4 were not adjusted for birth weight. Regression coefficients (β) for linear regression and adjusted odds ratio (AOR) for logistic regression, 95% confidence intervals (CI), R2 and *P-*values are presented. (^*^p-value < 0.05).

## Discussion

In this prospective cohort study of 616 pregnant women, higher third trimester maternal perceived stress and depressive symptom scores were associated with modest reductions in birth weight after adjustment for covariates but were not associated with gestational age. Neither perceived stress nor depressive symptom scores were significantly associated with the categorical outcomes of LBW or PTB. These findings suggest that maternal psychological distress may be associated with birth weight across the birth weight continuum without substantially increasing the risk of clinically defined adverse birth outcomes.

The prevalence of antenatal elevated stress (17.4%) and elevated depressive symptoms (21.4%) in this study was lower than the pooled global estimates of 52 and 28%, respectively, reported in a recent systematic review (11). This divergence may reflect variability in screening tools, gestational timing of assessment, and sociocultural context across studies (32–34). Importantly, 98% of women reported receiving social support from family and friends, which may have contributed to the lower prevalence of psychological distress. This is consistent with evidence that perceived social support buffers psychological stress and is inversely associated with antenatal depressive symptoms and perceived stress (35–37). However, self reported social support should be interpreted cautiously because social desirability bias cannot be excluded, particularly in collectivist societies (38, 39).

Experimental and epidemiological studies suggest that antenatal psychological distress can adversely influence fetal development through fetal programming, increasing the risk of impaired fetal growth and adverse health outcomes later in life (4, 5). Although previous systematic reviews have linked antenatal perceived stress and depressive symptoms with LBW and PTB (12, 15–17), our findings showed that higher third trimester maternal perceived stress and depressive symptom scores were associated with reduced birth weight but not with PTB. Biologically, psychological distress activates the HPA axis, increasing maternal cortisol levels and reducing placental 11β-hydroxysteroid dehydrogenase activity, thereby increasing fetal cortisol exposure and impairing placental nutrient transport (13). In addition, psychological distress may induce low grade inflammation and reduce uteroplacental blood flow, restricting fetal growth without necessarily triggering preterm labor. These mechanisms may explain the observed association with lower birth weight in the absence of an association with PTB.

Consistent with these mechanisms, higher third trimester perceived stress and depressive symptom scores were associated with modest reductions in birth weight (5.84 g and 7.45 g, respectively), but not with gestational age, LBW, or PTB. Similar findings have been reported by Gelaye et al. (40), who observed a 23.42 g reduction in birth weight without increased odds of LBW or PTB. In contrast, an earlier study reported a larger reduction in birth weight (55.03 g) and increased odds of LBW (1.32 fold) (41). The smaller effect observed in our cohort may reflect the relatively low prevalence of psychological distress and the near universal social support reported by participants, which may have attenuated HPA axis activation and its downstream effects on fetal growth. Collectively, these findings suggest that antenatal psychological distress may be associated with birth weight across a continuum, although the effect size in this population was insufficient to increase the prevalence of LBW.

Although adequate antenatal care is generally associated with improved pregnancy outcomes (42–44), we observed a higher prevalence of LBW among women with more antenatal visits and a significant association between cesarean delivery and PTB. These findings likely reflect underlying pregnancy complications rather than adverse effects of care, as women with high risk pregnancies typically require more frequent antenatal monitoring and are more likely to undergo medically indicated early delivery, a pattern consistently reported in obstetric literature (45).

The findings have important implications for antenatal care and support consideration of maternal psychological distress assessment as part of comprehensive antenatal care, particularly from a maternal wellbeing perspective. Although the observed association with birth weight was modest, early identification of psychological distress and appropriate psychosocial support may improve maternal well being and potentially contribute to better pregnancy outcomes. Future studies should investigate the biological role of social support in buffering HPA axis activation and modulating stress related biomarkers, such as salivary or hair cortisol and inflammatory markers, during pregnancy. Such studies could provide mechanistic insights into how psychosocial factors are related to fetal growth and help identify potential targets for interventions aimed at improving maternal and birth outcomes.

The strengths of this study include its prospective cohort design, validated screening scales for psychological distress, hierarchical gestational age ascertainment (with first trimester ultrasonography for most participants), and objectively recorded birth outcomes. Adjustment for relevant maternal and obstetric covariates further improved the robustness of the findings. A major limitation of our study is that maternal perceived stress and depressive symptoms were assessed only once during the third trimester using self reported screening scales, which may not reflect changes earlier in pregnancy or throughout gestation. Although analyses were adjusted for several maternal and obstetric factors, residual confounding cannot be excluded. In addition, the study was conducted in selected lower income urban communities, which may limit the generalizability of the findings. Approximately 14% of data were missing due to participant dropout or unavailability during perceived stress and depressive symptoms assessment. However, baseline characteristics were comparable between participants with and without these data, suggesting minimal attrition bias. Recruitment during the COVID-19 pandemic (2021–2022) may also have influenced maternal psychological wellbeing and access to healthcare, although none of the participants reported a history of COVID-19 infection; therefore, broader pandemic-related effects cannot be excluded. In addition, several potential confounders, including maternal BMI/nutritional status, gestational weight gain, were unavailable, limiting adjustment for residual confounding. Information on antidepressant medication use was also not collected from participants.

## Conclusion

Third trimester maternal perceived stress and depressive symptom scores were associated with modest reductions in birth weight but not with gestational age, LBW, or PTB in this population. These findings underscore the importance of integrating maternal mental health into antenatal care, not only for psychological well being but also for its potential influence on downstream biological pathways that may affect fetal growth and development. Further large scale longitudinal studies are warranted to better elucidate these relationships and the underlying mechanisms linking maternal mental health to fetal development.

## Data Availability

The raw data supporting the conclusions of this article will be made available by the authors, without undue reservation.
